# Trends and Changes in Treating Proximal Humeral Fractures in Italy: Is Arthroplasty an Increasingly Preferred Option? A Nation-Wide, Population-Based Study over a Period of 22 Years

**DOI:** 10.3390/jcm13195780

**Published:** 2024-09-27

**Authors:** Enrico Ciminello, Andrea Modesti, Emilio Romanini, Stefano Lepore, Gabriele Tucci, Stefano Di Gennaro, Giandomenico Logroscino, Paola Ciccarelli, Tiziana Falcone, Marina Torre

**Affiliations:** 1 Italian Implantable Prostheses Registry, Italian National Institute of Health, 00161 Rome, Italy; paola.ciccarelli@iss.it (P.C.); tiziana.falcone@iss.it (T.F.); marina.torre@iss.it (M.T.); 2 Department of Life, Health and Environmental Sciences, University of L’Aquila, 67100 L’Aquila, Italy; andrea.modesti@graduate.univaq.it (A.M.); giandomenico.logroscino@univaq.it (G.L.); 3 GLOBE, Italian Working Group on Evidence Based Orthopaedics, 00197 Rome, Italy; emilio.romanini@gmail.com (E.R.); tuccigab@yahoo.it (G.T.); 4 Polo Sanitario San Feliciano, 00166 Rome, Italy; stefanodige1@gmail.com; 5 I Orthopaedic Unit, A. Cardarelli Hospital, 80131 Naples, Italy; stefano.lepore@aocardarelli.it; 6 Ospedale dei Castelli, Ariccia, 00040 Rome, Italy; 7 Department of Minimally Invasive and Computer-Assisted Orthopaedic Surgery, University of L’Aquila, 67100 L’Aquila, Italy

**Keywords:** proximal humeral fractures, arthroplasty, shoulder, administrative data, epidemiology, registries, public health

## Abstract

**Background**: Proximal humeral fractures (PHFs) are common, especially in the elderly, and account for 4% to 10% of all fractures, with women more often affected than men. Treatments include conservative methods, internal fixation and arthroplasty, with surgical approaches increasingly being used due to technological advancements. This study analyzes the evolution of PHF treatments in Italy from 2001 to 2022, using data from the Italian Hospital Discharge Records (HDRs) Database, and includes a stratified analysis by age and sex. **Methods**: Using HDR data from 2001 to 2022, records with ICD9-CM codes for proximal humeral fractures (812.0 and 812.1) among diagnoses were selected and categorized into three treatment groups: arthroplasty, fixation and conservative. Time series were analyzed with stratification by sex and age. **Results**: The extracted data included 486,368 records of PHFs, with 223,742 cases treated surgically (arthroplasty or internal fixation) and 262,626 treated conservatively; the average patient age was 66.6 years, with a higher proportion of women, especially among arthroplasty patients. Over time, the use of fixation and arthroplasty increased from 20% of treatments in 2001 to over 60% in 2022, with fixation becoming the most common treatment method by 2014 and arthroplasty significantly increasing among women, particularly in the 65–74 and 75–84 age groups. **Conclusions**: The study shows that in Italy, over the past two decades, treatment for PHFs has shifted from conservative methods to a preference for internal fixation and increasingly for arthroplasty, particularly among women and patients aged 65–84, reflecting evolving trends and technological improvements.

## 1. Introduction

Proximal humeral fractures (PHFs) are very common and represent a serious health problem [1]. They are among the most frequent fractures in adults [2] and one of the most common age-related fractures in the elderly, with women more often affected than men. According to several studies conducted in different populations, prevalence ranges from 4 to 10% of all fractures [3]. PHFs account for about 80% of all humeral fractures. In general, they mostly occur after the age of 50 with low-energy trauma, whereas for patients younger than 50, high-energy trauma is involved, mainly in male patients [1]. It has been suggested that this rate increases as the average population age rises [4,5]. The incidence rate of these fractures varies considerably depending on the geographical area and the year of the study [6,7]. The treatment is sometimes controversial, and some cases may be technically challenging. Numerous studies have attempted to determine the optimal treatment for PHFs, but no definitive guidelines have been established, particularly in three- and four-part fractures and in the elderly population [8]. The difficulty lies mainly in finding the most appropriate treatment depending on the number of bone fragments and the involved areas of epiphysis [9,10]. The controversy between conservative and surgical treatment has intensified after the publication of the ProFHER trial [11]. Moreover, there is still considerable debate around comparisons of various operative modalities, mainly between intramedullary nailing, open reduction with internal fixation and arthroplasty, as to which is the best method to achieve better functional outcomes [12,13]. Indeed, several variables influence the choice of treatment, including age, bone quality, daily activities and other factors. Even the classification system for PHFs lacks consensus: while the Neer classification is globally the most widely used, other systems, such as those by Hertel or the AO system, are also applied [14,15]. Furthermore, for the same type of fracture, various fixation techniques and systems are frequently employed, leading to some confusion regarding the gold standard to be adopted [16]. Anyway, in recent years, the operative approaches seem to be increasingly used in many countries due to surgical and technological innovation.

The aim of this study is to describe how the approach to treating PHFs has evolved in the last two decades in Italy, with a particular focus on the differences in time trends of conservative treatment, fixation and arthroplasty, using population data from the Italian Hospital Discharge Records (HDRs) Database between 2001 and 2022. A stratified analysis by patient age and sex is also provided.

## 2. Materials and Methods

The Italian Ministry of Health consolidates the National Hospital Discharge Records (HDRs) Database annually and provides the Italian National Institute of Health with it. This data source allows the collection of administrative, demographic and clinical information on almost every hospitalization in the country, with coverage increasing from 94.2% in 2001 to 99% in 2019 [17]. Medical procedures performed during hospitalization and related diagnoses are reported in HDRs by using the ICD9-CM international classification coding system.

HDRs for the years from 2001 to 2022 were browsed, and only those records where at least one ICD9-CM code for diagnoses with the first four digits equal to 812.0 (Fracture of upper end of humerus closed) or 812.1 (Fracture of upper end of humerus open) appeared in one or more field of main or secondary diagnosis were selected. Records were then divided into three groups, based on ICD9-CM codes appearing in any of the main or secondary procedure fields, according to the procedure performed during hospitalization. If the code 81.80 (Total shoulder replacement) or 81.81 (Partial shoulder replacement) appeared, the patients were considered as treated via “Arthroplasty”. If the code 79.10 (Closed reduction of fracture with internal fixation), or 79.11 (Closed reduction of humeral fracture with internal fixation), or 79.30 (Open reduction of fracture with internal fixation), or 79.31 (Open reduction of humeral fracture with internal fixation) appeared, the patient was considered as treated with internal “Fixation”. If none of the above-mentioned codes appeared, the performed treatment was considered as “Conservative”.

The operative flow reporting the data extraction process is the following:

IF (812.0 OR 812.1) IN diagnoses (first four digits) THEN keep the record
IF (80.80 OR 80.81) IN procedures THEN record is arthroplasty
IF (79.10 OR 79.11 OR 79.30 OR 79.31) IN procedures AND (81.80 OR 81.81) NOT IN procedures THEN record is fixation
IF (79.10 AND 79.11 AND 79.30 AND 79.31 AND 81.80 AND 81.81) NOT IN procedures THEN record is conservative

This operative flow introduces by construct a hierarchy in the group assignment. Arthroplasty is at the highest level of the hierarchy, while conservative treatment is at the lowest one and fixation is in between. This respects the burden of the treatment on patients, with arthroplasty being the most invasive and definitive treatment, used when the others would have no or little efficacy, while conservative is the one with the lowest impact and fixation represents an intermediate approach. This hierarchy implies that, for instance, a record for which both arthroplasty and fixation codes appear would be labeled as arthroplasty, assuming that it was necessary after an ineffective fixation.

Once the records of interest were extracted, the time series were analyzed on an annual basis for overall surgical volume and stratified by sex and age class, according to the partition used by the Italian Arthroplasty Registry: Age < 45; 45 ≤ Age < 55; 55 ≤ Age < 65; 65 ≤ Age < 75; 75 ≤ Age < 85; Age ≥ 85 [18]. Both absolute counts and proportions were taken into account. The series were decomposed and variations in trends and proportions were investigated via the Cox–Stuart test and the Proportion Trend test, respectively. The threshold for statistical significance was fixed to 0.05. Statistical analysis was performed via software R version 4.2.3 (2023-03-15 ucrt)—“Shortstop Beagle”.

## 3. Results

**Figure 1:**
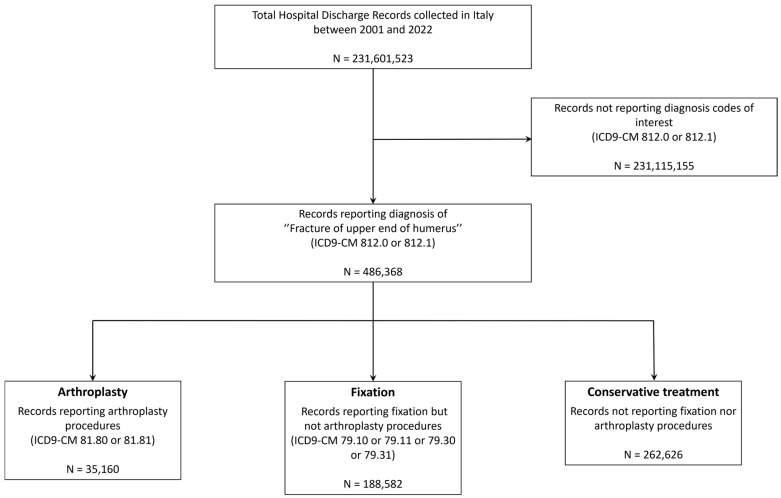
Data extraction flowchart.

The HDR Database included 231,601,523 records from 2001 to 2022. Out of these, 486,368 records were extracted as satisfying the condition on diagnoses involving fractures of the upper end of the humerus. In total, 223,742 cases were treated via invasive surgery (either arthroplasty or internal fixation) and 262,626 were treated with a conservative approach. The data extraction process is summarized in the flowchart in Figure 1.

The overall average age was 66.6 (21) years, with patients treated by arthroplasty being generally older than the other groups, with an average age of 73.5 (11.2) years. Patients were mainly females (70.3%), in particular among those treated with arthroplasty (82.5%). Patient distribution over sex and age classes is reported in Table 1.

**Figure 2:**
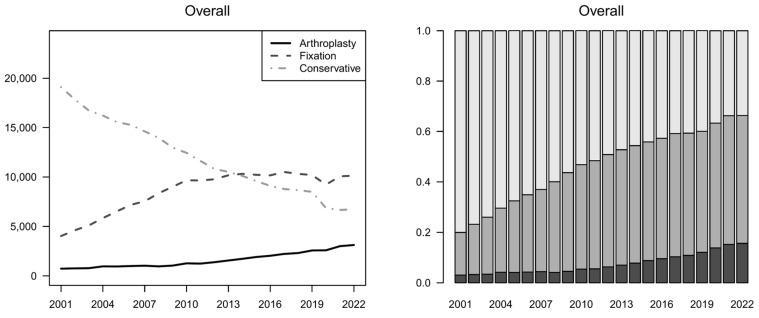

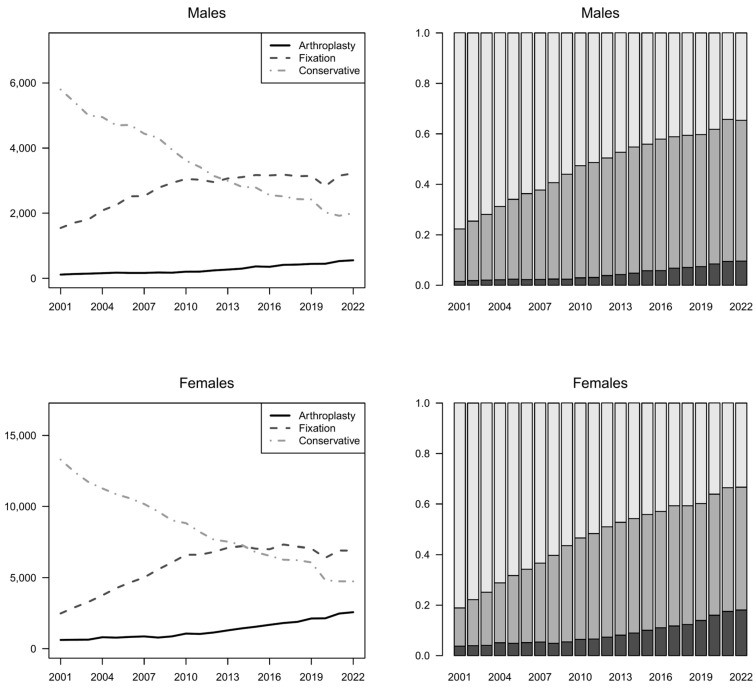
Trends by treatment group (2001–2022). Counts (**left** column) and proportions (**right** column), overall and by sex. Dark gray: arthroplasty; gray: fixation; light gray: conservative.

The number of hospital admissions considering the ICD9 codes of interest significantly decreased in the observed period (*p* < 0.01). The number of fractures treated with fixation and arthroplasty increased over time, both in absolute terms and proportion, shifting from 20% of the total treatments in 2001 to over 60% in 2022 (*p* < 0.01). The number of internal fixations increased until it exceeded the number of conservative treatments in 2014, and it remained the most used way to treat PHFs until 2022. When stratifying by sex, fixation became the most used technique for males in 2013 and for females in 2014. Moreover, arthroplasty strongly increased in females, accounting for 20% of the total choices for females (Figure 2 and Supplementary Materials).

**Figure 3:**
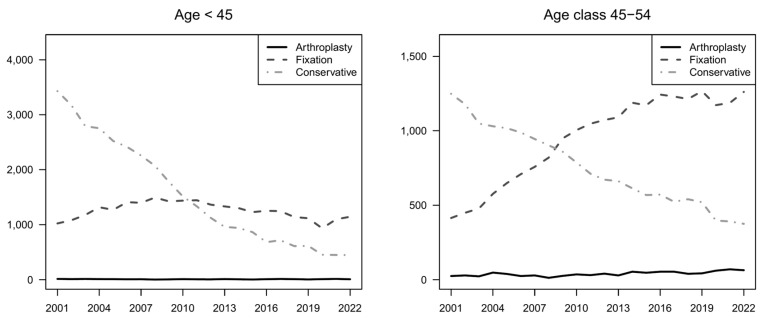

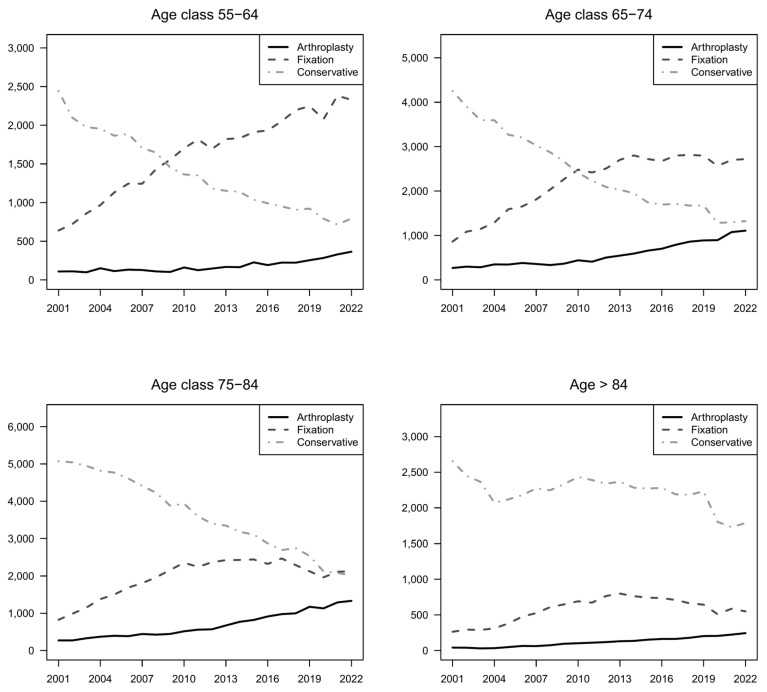
Trends by treatment group (2001–2022). Counts by age class.

**Figure 4:**
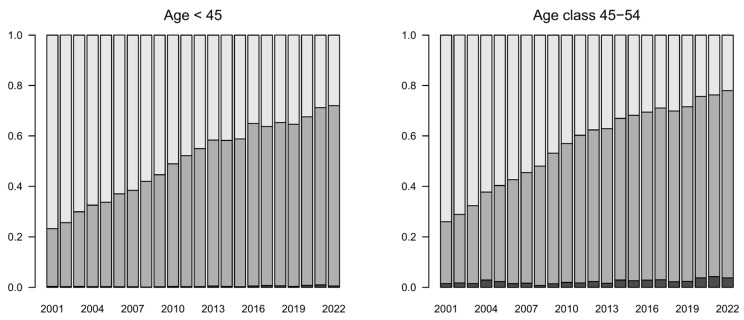

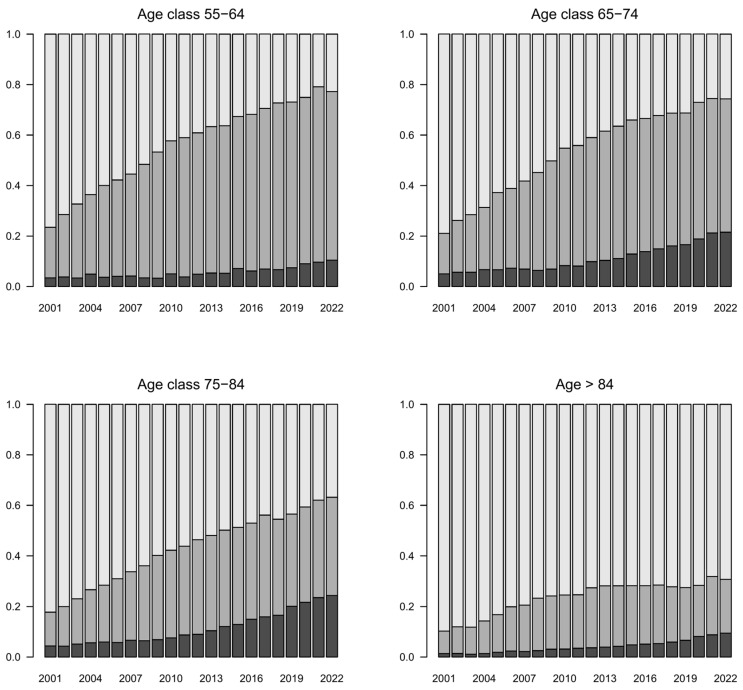
Trends by treatment group (2001–2022). Proportions by age class. Dark gray: arthroplasty; gray: fixation; light gray: conservative.

The age class analysis highlighted that the increase in preference towards arthroplasty was stronger in age classes 65–74 and 75–84 (Figure 3 and Figure 4 and Supplementary Materials). In general, this approach increased only slightly and is usually avoided in the youngest and oldest patients.

Fixation became the most performed treatment in all age classes except for the elderly, more precisely in the age class over 84 years, where the conservative approach was always the most preferred option (Figure 3 and Figure 4 and Supplementary Materials).

## 4. Discussion

This study targeted the analysis of the evolution of PHF treatment approaches in hospitalized patients over a period of 22 years (2001–2022) in Italy, mainly observing the differences in time trends for arthroplasty, fixation and conservative treatment in the Italian population with stratification by age and sex. The work highlighted how the approach to treating PHFs in Italy has changed over the last two decades. The use of arthroplasty has increased over time, and although it was the less used approach during the 22 years under consideration, it accounted for almost 20% of all treatment choices in 2022. Fixation has increased over time, remaining the preferred treatment during the observed period. Conservative treatment, which was the most used approach in 2001, has been decreasingly used over time and was overtaken by fixation in 2014. Arthroplasty is an increasingly selected option for females and for patients between 65 and 84 years of age. On the other hand, the choice of fixation spikes among the youngest, while a conservative approach is the preferred option for the elderly.

In recent years, there have been considerable improvements in surgical options for PHF treatment. Open reduction internal fixation with angular stability systems, minimally invasive plate osteosynthesis surgical approaches, 3D reconstruction models, computerized planning software, jig cutting guides, patient-specific instrumentation, robotic-assisted surgery and, more recently, artificial intelligence are the most notable advancements in PHF surgery [19,20]. The introduction of locking plates has broadened the possible indications for open reduction and internal fixation. This treatment option is favored, in particular for younger patients, for most fracture configurations, as it is suitable for four-part fractures and allows direct visualization of the reduction and grafting techniques [21,22]. The evolution of reverse total shoulder arthroplasty design [23] and the continuous improvement of the surgical technique have led to better and more reproducible outcomes, especially in complex fractures and in older patients [24]. Indeed, studies have shown that arthroplasty might provide a benefit for patients, even if more invasive and expensive than fixation. Nowadays, this surgical option may allow for a shorter recovery time and a better functional restoration [13], especially in the 64–75 age group where the use of this technique has significantly increased. Furthermore, arthroplasty is preferred in women over 65 years of age compared to men, probably due to the higher bone fragility that would cause less successful fixation [25]. On the other hand, arthroplasty is less used in younger patients because of the naturally limited device survival, which could potentially lead to multiple revisions during the lifespan of the patients. At the same time, for patients over 85, this surgical procedure may have a higher impact on their general health because of the high surgical risk. However, based on the recent results of a Cochrane Systematic Review [26], it seems that there is evidence of no differences between surgical and non-surgical treatment in patient-reported function and quality of life. Moreover, there is no clear indication of which treatment is preferable, considering that surgery compared with non-surgical treatment showed high or moderate evidence that it does not have a better outcome at one and two years after injury.

The results of the current study confirm the trend observed in Germany until 2016, where the operative treatment of PHFs by fixation and arthroplasty increased [27]. On the other hand, they contrast with the findings in Australia, Finland and the USA, where the non-operative approach is increasingly preferred together with arthroplasty, in particular in patients over 65 and in females, whereas the fixation approach is decreasingly used because of the risk of postoperative complications [28,29,30,31]. Further studies at the international level, providing population data and a wide variety of metrics (socioeconomics, health, sport and diet habits, environmental), may investigate differences in incidence between countries, injury patterns and reasons behind the choices of treatment. At present, the lack of evidence may lead to different approaches and clinical practices being followed in different countries.

To our knowledge, this is the first population study performed in Italy on PHFs and related treatments at the population level and over such a long period of time. This study therefore offers a global view of the PHF phenomenon and shows how its treatment has evolved over 22 years. Furthermore, the analysis by sex and age gives a clear picture of the evolution of this issue in the population by its characteristics.

The main limitation of this study lies in the administrative nature of the data used. The diagnosis and related treatment procedure were identified by using the ICD-9 ICM coding, which could be subject to error due to the incorrect compilation of the source data and the lack of an audit on the accuracy and clinical significance of the codes reported. Indeed, the ICD9-CM codes for arthroplasty only distinguish between total and partial shoulder replacement and cannot identify revisions. This may result in a bias of analyzed counts and does not allow for further studies on device survival and the subsequent efficacy and safety of arthroplasty in the treatment of PHF. Moreover, ICD9-CM codes do not provide technical information on the device, like the design (anatomical or inverse), making any discrimination impossible in such sense. Therefore, the use of such administrative data is useful to provide an overview of the epidemiological patterns in hospitalized patients, but caution should be recommended when drawing a conclusion of clinical relevance. A further possible limitation of this study is related to the fact that, due to the intrinsic nature of the data collected, we only evaluated patients with PHFs requiring hospitalization, excluding from the analysis those treated conservatively in outpatient care. As only a small percentage of patients intended for conservative treatment are admitted to hospital, this introduces a potential bias regarding the real number of surgical and conservatively treated patients. Another limitation is that this system lacks a classification of fractures and does not provide information on the severity of fractures, which certainly influences the indication to treatment.

HDRs do not report any information on design, materials of the devices or perioperative information (e.g., operated side and approach), which makes it difficult to carry out a complete assessment of arthroplasties. For this reason, given the increasing trend in arthroplasty, in particular for patients between 65 and 84 years of age, a dedicated tool for data collection, focusing on meaningful information about the devices and the procedures, is of primary importance. To this purpose, the Italian Arthroplasty Registry (Registro Italiano Artroprotesi, RIAP [32]), established by law at the Italian National Institute of Health (Istituto Superiore di Sanità, ISS, Rome, Italy), has been collecting records about shoulder replacements with a specifically implemented data collection flow since 2017. The RIAP registry, like all medical device registries, is an extremely powerful and important tool for monitoring the long-term safety and efficacy of devices, thereby contributing to the protection of patient health. As proved by arthroplasty registries worldwide, such tools allow the long-term tracking of devices, enabling the early identification of potential safety problems, subsequently enhancing product recalls if necessary, and allow studies with high detail on important clinical issues. For instance, registries make it possible to perform dedicated studies analyzing devices’ design and technical information, which is essential for deriving epidemiological and clinical conclusions to improve patients’ health and decision-making. However, registries need to be continuously fed with complete and representative data on the phenomenon, which is not yet possible for RIAP, given the current voluntary nature of its feeding [33,34].

## 5. Conclusions

The study highlights that over the past two decades in Italy, there has been a significant shift in the treatment of PHFs in hospitalized patients, with a marked increase in the use of surgical methods, particularly fixation and arthroplasty, which has increased from 20% to over 60% of treatments. This trend is especially evident among older patients and women, with fixation becoming the leading treatment method since 2014. The preference for arthroplasty has grown notably in the 65–84 age groups, indicating an evolving approach towards more invasive treatments over time due to technological advancements and improved outcomes.

The significant increase in the number of joint replacements worldwide and the lack of evidence for the treatment of PHFs highlight the crucial need to promote the establishment of topic-specific registries to collect data prospectively on shoulder arthroplasty revision surgery [24]. Indeed, the development of new technologies brings new prostheses with new designs onto the market, and registries should be able to record as many features as possible to properly monitor devices and produce reliable assessments.

To achieve this aim, the International Society of Arthroplasty Registries (ISAR [35]) paved the way to forge common agreements at the international level within the arthroplasty registry community [36]. The Orthopaedic Data Evaluation Panel (ODEP), an independent panel of experts, provides objective evaluation of the evidence reliability of medical implants performance. It has developed a system to assess shoulder prostheses up to 10 years survival and has already evaluated 154 items related to shoulder replacement [37].

A synergic interaction between these different infrastructures may provide sound evidence for a better and safer treatment of patients, which should be reflected in an improvement in patient safety.

## Figures and Tables

**Table 1:** Patients’ features by treatment group.

	Arthroplasty	Fixation	Conservative	Total
N	35,160	188,582	262,626	486,368
Age	73.5 (11.2)	63 (19.2)	68.3 (22.8)	66.6 (21)
Females	29,001 (82.5%)	128,213 (68%)	184,694 (70.3%)	341,908 (70.3%)
Males	6159 (17.5%)	60,369 (32%)	77,932 (29.7%)	144,460 (29.7%)
Age < 45	219 (0.6%)	27,629 (14.7%)	33,900 (12.9%)	61,748 (12.7%)
44 < Age < 55	881 (2.5%)	20,941 (11.1%)	16,541 (6.3%)	38,363 (7.9%)
54 < Age < 65	3925 (11.2%)	35,796 (19.0%)	30,324 (11.5%)	70,045 (14.4%)
64 < Age < 75	12,468 (35.5%)	48,448 (25.7%)	53,497 (20.4%)	114,413 (23.5%)
74 < Age < 85	15,055 (42.8%)	43,143 (22.9%)	79,388 (30.2%)	137,586 (28.3%)
Age > 84	2612 (7.4%)	12,625 (6.7%)	48,976 (18.6%)	64,213 (13.2%)

## Data Availability

The data presented in this study are available in aggregated form on request from the corresponding author.

## Supplementary Materials

The following supporting information can be downloaded at https://www.mdpi.com/article/10.3390/jcm13195780/s1. Supplementary_File_S1.xlsx contains the counts of the following time series by year on which figures in this manuscript are built: total Proximal Humeral Fractures (PHFs); PHFs treated by arthroplasty, fixation and conservative approach; PHFs by sex; PHFs by age class.
